# Characterization of ESBL (SHV-12) producing clinical isolate of *Enterobacter aerogenes *from a tertiary care hospital in Nigeria

**DOI:** 10.1186/1476-0711-9-1

**Published:** 2010-01-12

**Authors:** Murat Kasap, Kayode Fashae, Sinem Torol, Fetiye Kolayli, Fatma Budak, Haluk Vahaboglu

**Affiliations:** 1 Tibbi Biyoloji AD, KOU Tip Fakultesi, Izmit, Turkey; 2 Department of Botany and Microbiology University of Ibadan, Nigeria; 3 Mikrobiyoloji ve Klinik Mikrobiyoloji AD, KOU Tip Fakultesi, Izmit, Turkey; 4 Enfeksiyon Hastaliklari ve Klinik Mikrobiyoloji AD, KOU Tip Fakultesi, Izmit, Turkey

## Abstract

**Background:**

We studied the beta-lactamases of an *E. aerogenes *isolate recovered from the blood of a two-year-old patient. The isolate demonstrated a disk-diffusion phenotype typical for an AmpC-ESBL co-producer.

**Methods:**

Microbiology studies were performed according to standard protocols. The resistance gene was identified by transconjugation and cloning experiments.

**Results:**

By transconjugation only a narrow spectrum beta-lactamase (TEM-1) encoded on a small plasmid was transmitted. The ESBL was cloned and expressed in an *E. coli *host. Sequence analysis of the recombinant plasmid revealed *bla*_SHV-12 _associated to the insertion sequence, IS*26*.

**Conclusion:**

This is the first study demonstrated the occurrence of SHV-12 in Nigeria.

## Background

Enterobacter species, in particular, *E. cloacae *and *E. aerogenes *are able to compromise antibacterial treatment by over expressing the chromosomal AmpC beta-lactamase [1,2]. Emergence and spread of Class A extended-spectrum beta-lactamases (ESBLs) among these species are further complications [3].

ESBLs confer resistance to expanded-spectrum beta-lactam antibiotics. The majority of these enzymes are derived by amino acid substitutions from the narrow spectrum precursors, TEM-1, 2 and SHV-1. TEM-type ESBLs generally disseminate on transposons, Tn1, Tn2 and Tn3 [4]. SHV-type ESBLs, on the other hand, are typically associated to *IS*26 and disseminate through *IS26 *dependent mobilization events from the *K. pneumoniae *chromosome [5,6]. Eventually, the extended-spectrum derivates of TEM and SHV enzymes are now ubiquitous.

We detected an ESBL producing *E. aerogenes *clinical isolate from Nigeria. Since, the data in the literature regarding the occurrence and the dissemination of ESBLs in Nigeria is limited [7,8], we characterized the beta-lactamases of this isolate by microbiological and molecular means

## Methods

### The strain and the susceptibility tests

A strain of *Enterobacter aerogenes *was isolated and identified by standard methods from the blood of a two-year-old male patient, admitted (8th November 1999) to a tertiary care hospital in a Southwestern city of Nigeria, with clinical diagnosis of febrile convulsion. The case note is, however, not available for the history and outcome of the patient. The strain was identified by API 20E (bioMérieux Marcy l'Etoile, France) according to the instructions provided by the manufacturer.

Disk diffusion test was performed on Mueller-Hinton agar (disks and agar media were from Oxoid, Basingstoke UK) for the phenotypic identification of ESBLs as described elsewhere [9]. Briefly, the cefotaxime (CTX; 30-μg) disk was placed 20 mm away from the amoxicillin (20-μg)-clavulanate (10-μg) (AMC) disk, the ceftazidime (CAZ; 30-μg) disk was placed at 30 mm distance, and the cefepime (FEP; 30-μg) disk was placed at 30 mm distance. For the phenotypic detection of the AmpC enzyme, a cefoxitin (FOX; 30-μg) disk was placed on the agar, as well.

The MICs of key antibiotics were determined by the broth micro-dilution test using Mueller-Hinton Broth (Oxoid, Basingstoke UK) as recommended by CLSI. End-points were interpreted after 18 h of incubation at 37°C. *E. coli *ATCC 25922 and *E. coli *DH10B were included as control strains. Powder forms of antibiotics were obtained from local companies: ampicillin (Mustafa Nevzat), piperacillin & tazobactam (Wyeth), clavulanate (DEPA), cefepime (Bristol-Myers Squibb), cefotaxime (Toprak), ceftazidime (Glaxo-SmithKline), imipenem (Merck), meropenem (Astra-Zeneca), ciprofloxacin (Bayer), gentamicin (Bilim) and tobramycin (Nobel). The final concentration of clavulanate was 4 mg L^-1^.

### Plasmid studies

Plasmids were isolated by the alkaline lysis or the Kado Liu methods [10], run on 0.8% agarose gels and visualized under UV light. Transconjugation and transformation experiments were performed with *E. coli *J53-2 (Rif^R^) and electro-competent *E. coli *DH10B strains as recipients, respectively [11,12].

### DNA & RNA isolation, PCR and RT-PCR

DNA templates for PCR experiments were prepared by simply boiling a dense bacterial suspension in ddH_2_O and a 10 min of centrifugation at 16.000 × g. DNA-free RNAs were isolated with RNeasy Mini Kit (Qiagen), and run on denaturating gel conditions to check the integrity of RNAs and the lack of visible DNA contamination. cDNAs were immediately synthesized by random hexamer primers with Revert Aid first strand cDNA synthesis kit (Fermentas, Lithuania).

PCR reactions were set in 50 μl final volumes made up of 1× buffer, 1.5 U Taq polymerase (Fermentas, Lithuania), 1.5 mM MgCl_2_, 0.8 mM dNTPs, 50 pmol primers each. RT-PCR was set up with the same master mixture except, 1× SYBR Green I (Sigma) was added. Reactions were prepared on ice and run on Quantica (Techne) RT-PCR Thermal cycler as described elsewhere [13]. Specificity of the product was assessed by the dissociation curve analysis made by the Quantica software and the relative mobility of the PCR products on the agarose gels.

Primers, ShvF1-5'-ATTACCATgAgCgATAACAg-3' and ShvR1-5'-CATTCAgTTCCgTTTCCC-3' were used (55°C annealing temperature) in RealTime-PCR to amplify a 133 bp fragment internal to *bla*_SHV-12 _gene. The primer ShvF1 and the primer DeoRR1-5'-CCAggTggTCACCAATgATT-3' were used (50°C annealing temperature) to amplify a 927 bp fragment which extends from the 3' end of the *bla*_SHV-12 _gene to the 402 bp of the transcriptional regulator gene. To amplify the entire sequence (861 bp) of *bla*_TEM _gene, the primers TemA-5'-ATgAgTATTCAACAT TTCCgTg-3' and TEMD-5'-TTACCAATgCTTAATCAgTgAg-3' (annealing temp. 52°C) were used.

### IEF

Crude cell extracts were prepared by sonication. Analytical isoelectric focusing (IEF) was performed on a 5% polyacrylamide gel containing ampholytes (pH range, 3-10; Bio-Rad Laboratories, USA) with a Model 111 Mini IEF Cell (Bio-Rad). SHV-1 (pI 7.6), OXA-14 (pI 6.2) and TEM-1 (pI 5.4) were included as references. Enzymes were focused at a constant 1 W for 45 min and detected by overlaying the gel with 1 mM nitrocefin solution.

### Cloning and sequencing

High molecular weight (HMW) genomic DNA was isolated using the procedure described by Chen and Kuo [14]. For plasmid DNA isolation, alkaline lysis method was used (Sambrook and Russell, 2001). Twenty micrograms each of HMW genomic DNA and plasmid DNA were digested with 0.1 units of *Bsp*143I (Fermentas) for 20 min at 37°C and 0.5 microgram of the cloning vector, pZero (Invitrogen), was digested with 5 units of *Bam*HI (Fermentas) for one hour at 37°C. Digested DNA and the vector were phenol/chloroform extracted and compatible arms were ligated overnight at 16°C. Two microliters of ligation mix was then transformed to electrocompetent *E. coli *DH10B and the recombinant clones were selected on agar plates supplemented with ampicillin (100 mcg/mL) plus zeocin (50 mcg/mL).

Dye terminator cycle sequencing with the ABI Prism BigDye Terminator kit (Applied Biosystems, Foster City, Calif.) were used to obtain the sequences. The assay was carried out according to the standardprotocol. Data was collected on an ABI 377 automated fluorescence sequencer.

## Results

The isolate, identified as *E. aerogenes *(*Ea*N146), exhibited a phenotype by the disk diffusion test that is typical for an AmpC-ESBL co-producer [9]. Briefly, there was no zone around the FOX disk which was specifically caused by the over-expressed AmpC enzyme whereas the zone around the FEB disk was enlarged on the AMC side, indicating the existence of a Class A ESBL.

By transformation we were able to transfer a narrow spectrum enzyme encoded on a <7 kb cryptic plasmid (data not shown) to DH10B recipient (TN146) which was identified as TEM-1 by sequencing on both sides of the PCR product.

**Figure 1:**
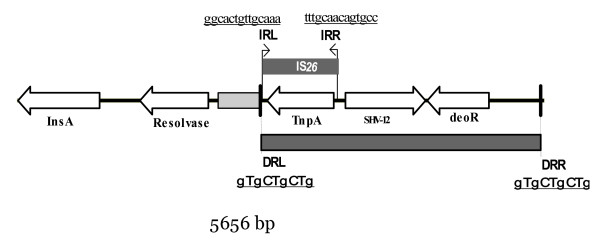
**The schematic map of the cloned insert (5656 bp)**. ORFs are indicated by empty arrows and repeat regions by dark shaded boxes.

By shotgun cloning, on the other hand, we recovered a positive clone (CN146) possessing an ESBL. Sequencing of the recombinant plasmid (pCEaN146) from this clone revealed a 5656-bp insert (Figure 1; GenBank accession # EU534207) on which we determined an open reading frame (ORF) encoding the ESBL, SHV-12 and an insertion sequence, *IS26*, upstream (73-bp) of the *bla *gene with perfect 14 bp terminal inverted repeats [15]. With the aid of ORF finding software (MB DNA Analysis v6.84) and Blast comparison against the sequences submitted to GenBank we identified three more intact ORFs and one another ORF disrupted by *IS26*. Interestingly, we detected a 9-bp direct repeat sequence (GTGCTGCTG) flanking on the left and right boundaries of a 3002-bp which might be an incomplete transposable unit.

MICs of the strains are shown in Table 1. *E. aerogenes *is highly resistant to beta-lactam antibiotics except carbapenems. In this strain clavulanate apparently potentiated the activity of cefepime which is relatively stable against the AmpC type enzyme and hydrolyzed only by the ESBL, but not enhanced the activity of ceftazidime which is hydrolyzed by the clavulanate resistant AmpC enzyme [9].

**Table 1:** The MICs of key antibiotics for SHV-12 producing *E. aerogenes *(*Ea*N146), the transformant (TFN146) and the SHV-12 producing clone (CN146)

Ab^a^	μg/ml		
	
	*Ea*N146	TFN146	CN146
Ampicillin	≥256	≥256	≥256
Ampicillin/Clav	≥256	64	32
Cefepime	16	0,5	128
Cefepime/Clav	0,025	0,025	0,025
Ceftazidime	128	0,5	≥256
Ceftazidime/Clav	64	0,5	4
Cefotaxime	16	<1	>256
Piperacillin	32	>128	>128
Piperacillin/Tazo	<4	<4	>128
Imipenem	<1	<1	<1
Meropenem	<0.25	<0.25	<0.25
Ciprofloxacin	<0.25	<0.25	<0.25
Gentamicin	>16	<1	<1
Tobramycin	8	<1	<1

^a^Clav, clavulanate (4 μg/ml), Tazo, tazobactam (8 μg/ml)

**Figure 2:**
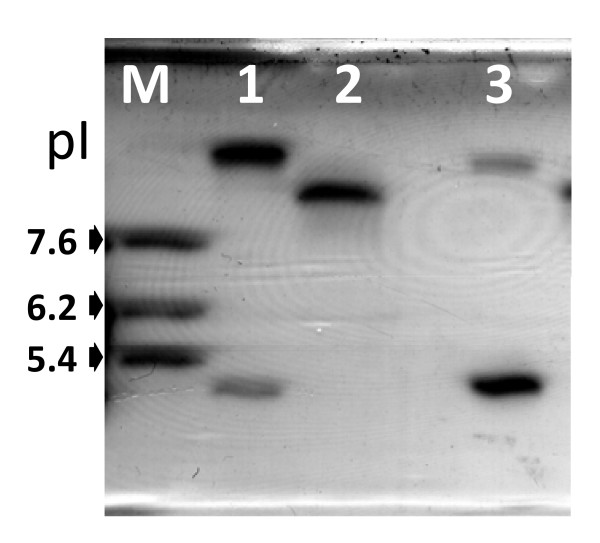
**IEF in ampholyte gradient of pH 3 to 10 with crude extracts**. Lane 1, enzymes (pIs, 5.4 & ≈ 9.0) from E. aerogenes (EaN146); lane 2, enzyme (pI 8.2) from the clone (CN146) and lane 3, enzymes (pIs, 5.4 & ≈ 8.9) from the transformant (TN146). The enzymes at pI 9 in lane 1 and pI 8.9 in lane 3 represented chromosomal enzymes (AmpC).

The IEF experiment is presented in Figure 2. Only two enzymes (pI 5.4 & pI 9) were visible in the lane of *E. aerogenes*, one representing the AmpC (pI 9) and the other representing the TEM-1 (pI 5.4). At pI 8.2, after several minutes of incubation a faint band, possibly corresponding to SHV12, appeared while the other bands diffused preventing us to record a good image (data not shown). To confirm the expression of SHV-12 in *Ea*N146, we performed a RT-PCR experiment with primers ShvF1 and ShvR1. Template DNAs were transcribed from the mRNAs of *Ea*N146, CN146 and TN146. This experiment demonstrated that, although low in amount, *Ea*N146 is expressing SHV-12.

## Discussion

This study characterized the beta-lactamases from a clinical isolate of *E. aerogenes *and demonstrated the occurrence of IS*26 *associated *bla*_SHV-12 _in Nigeria. SHV-12 was first identified in 1997 in Switzerland and later reported from various continents including Africa [16-20]. Reports of SHV-12 producing *E. cloacae *and *Klebsiella *blood isolates from Tanzania and Cameroon and now *E. aerogenes *from Nigeria indicate a high endemicity of SHV-12 possessing Enterobacteriaceae in the Western coast of Africa and therefore attract interest.

SHV-1 is supposed to be a species specific enzyme encoded mainly on the chromosome of *K. pneumoniae *[5,21]. Evolutionary analysis of submitted sequences indicated that extended-spectrum variants evolved on two branches from *bla*_SHV-1_, both mediated by IS*26 *depended mobilization events from the chromosome of *K. pneumoniae *[6]. These analyses revealed that *bla*_SHV-12_evolved from the branch of *bla*_SHV-2a_. Indeed, the genes surrounding *bla*_SHV-12 _in this study is identical to the genes surrounding *bla*_SHV-2a _in several previously submitted sequences (GenBank accession #s: X84314 and X53817). This suggests that *bla*_SHV-12 _identified in this study has evolved from *bla*_SHV-2a _as supposed by the evolutionary approach [6].

IS*26 *is an 820-bp long insertion sequence that typically generates 8 bp target duplication upon transposition [15]. It is demonstrated for IS*1 *that the length of target site duplication sequences may vary according to the sequence of the integrated site [22]. This phenomenon has not been studied for IS*26*. In this study we identified a 9 bp direct repeat on the boundaries of a 3002 bp region bearing *bla*_SHV-12 _and the DeoR type regulator gene. This 3002 bp region may be a replicating unit.

Data for ESBLs from Nigeria are rare in the literature [7,8,23]. In a study by Soge et al., CTX-M-15 producing *K. pneumoniae *clinical isolates were characterized [7]. In another study, the authors characterized ESBLs by phenotypic means among *E. aerogenes *[8].

## Conclusions

This is the first study reporting the occurrence and the genetic support of ESBL *bla*_SHV-12 _gene in Nigeria.

## Competing interests

The authors declare that they have no competing interests.

## Authors' contributions

The microbiology work including the resistance tests and tansconjugation was performed by KF, FK and FB. Molecular biology work including cloning and sequence analysis of the clone was performed by MK and ST. The study design, preparation of the manuscript and all aspects of intellectual contributions were by MK and HV. All authors read and approved the final manuscript.
